# Functional complementation of *Leishmania* (*Leishmania*) *amazonensis* AP endonuclease gene (*lamap*) in *Escherichia coli* mutant strains challenged with DNA damage agents

**DOI:** 10.1590/0074-02760150412

**Published:** 2016-05

**Authors:** Erika Verissimo-Villela, Milene Yoko Kitahara-Oliveira, Ana Beatriz de Bragança dos Reis, Rodolpho Mattos Albano, Alda Maria Da-Cruz, Alexandre Ribeiro Bello

**Affiliations:** 1 Universidade do Estado do Rio de Janeiro, Faculdade de Ciências Médicas, Departamento de Microbiologia, Imunologia e Parasitologia, Rio de Janeiro, RJ, Brasil; 2 Fundação Oswaldo Cruz, Instituto Oswaldo Cruz, Laboratório Interdisciplinar de Pesquisas Médicas, Rio de Janeiro, RJ, Brasil; 3 Universidade do Estado do Rio de Janeiro, Instituto de Biologia Roberto Alcântara Gomes, Laboratório de Genoma, Rio de Janeiro, RJ, Brasil

**Keywords:** AP endonuclease, Leishmania amazonensis, lamap, Base Excision Repair, DNA repair

## Abstract

During its life cycle *Leishmania* spp. face several stress conditions that can cause DNA damages. Base Excision Repair plays an important role in DNA maintenance and it is one of the most conserved mechanisms in all living organisms. DNA repair in trypanosomatids has been reported only for Old World *Leishmania* species. Here the AP endonuclease from *Leishmania (L.) amazonensis* was cloned, expressed in *Escherichia coli* mutants defective on the DNA repair machinery, that were submitted to different stress conditions, showing ability to survive in comparison to the triple null mutant parental strain BW535. Phylogenetic and multiple sequence analyses also confirmed that LAMAP belongs to the AP endonuclease class of proteins.

Unlike other trypanosomes, parasites of *Leishmania* genus are able to multiply within phagolysosomes (Murray 1981, Pearson & Steigbigel 1981, Thi et al. 2012) and survive to reactive oxygen species such as hydrogen peroxide (H_2_O_2_), nitric oxide (NO) and other potential damaging agents typically found in these organelles. This sort of reactive oxygen species as well as alkylating agents such as aminofluorene (AF) or methyl methane sulfonate (MMS) are responsible for changes of various macromolecules within the cell. Most of the researchers attention has been focused on damaging induced in DNA, which inhibits gene expression and stops the replication process. These lesions generate alkylating or oxidative species and yield AP sites (apurinic or apyrimidinic) that impair the information contained in a purine or pyrimidine bases from the deoxyribose backbone of DNA (Kow 2002).

The AP sites are repaired by a mechanism known as Base Excision Repair (BER), performed by enzymes of the AP endonuclease family (Demple & Harrison 1994), as well as by DNA glycosylases, DNA polymerase and DNA ligases (Doetsch & Cunningham 1990). There are two major AP endonucleases families firstly described in *Escherichia coli*: exonuclease III (EXO III) and endonuclease IV (ENDO IV) encoded by the *xth* and *nfo* genes, respectively (Cunningham et al. 1986, Barzilay & Hickson 1995). Endonuclease III (ENDO III), encoded by the *nth* gene, has also been described in *E. coli* and performs the repair of AP sites with an AP lyase function (Cunningham & Weiss 1985, Thayer et al. 1995). Besides AP endonuclease activity, APE1 presents a variety of functions involving DNA repair such as 3’ to 5’ exonuclease, 3’- repair diesterase as well as damaged RNA cleavage and multiple transcription regulatory roles (Li & Wilson 2014).

Till the present time, there are a few reports in the literature concerning enzymatic mechanisms involved in DNA repair in trypanosomatids and, most of them if not all, when dealing with *Leishmania* species are focused on the Old World ones (Passos-Silva et al. 2010). cDNA clones of *Trypanosoma cruzi* and *L.* (*L.*) *major* encoding DNA repair enzymes with a striking sequence similarity to EXO III (Pérez et al. 1999) conferred resistance to alkylating as to oxidative agents in *E. coli* strains that were deficient in EXO III and ENDO IV activities. Vidal and co-workers made a crystal structure of *L. major* AP endonuclease and compared to human APE1 (also known as HAP1 or Ref1). These authors demonstrated that the *L. major* enzyme featured apurinic/apyrimidinic endonuclease activities of the same magnitude as the eukaryotic and prokaryotic counterparts, displaying also important 3′-phosphodiesterase activity. LMAP efficiently repairs apurinic/apyrimidinic sites generated by alkylating agents such as MMS and 3′-blocked termini, as result of DNA single-strand breaks generated by oxidative molecules like H_2_O_2_ in *E. coli* repair-deficient mutants. In contrast, the expression of the human homologue only reverts MMS suceptibility (Vidal et al. 2007).

The AP endonuclease gene from *Trypanosoma brucei* was identified and deleted revealing that the parasites became hypersensitive to DNA lesions induced by different agents like methotrexate and phleomycin (Charret et al. 2012). In another study carried out by Furtado and co-workers (Furtado et al. 2012) the 8-oxoguanine DNA glycosylase 1 from *T. cruzi* (TcOgg1) was capable of complementing the activity of an Ogg1-defective *Saccharomyces cerevisiae* strain. They also demonstrated that the overexpression of TcOGG1 in *T. cruzi* led to different growth rates under non-stressed and H_2_O_2_ oxidative stressed conditions (Furtado et al. 2012). More recently, the results obtained from an approach where the *T. cruzi* and *T. brucei* MSH2 DNA mismatch repair protein was knocked out indicate that in both trypanosomes, in addition to its role as a key component of MMR, MSH2 is also directly involved in the response to oxidative stress (Grazielle-Silva et al. 2015).

Moreover, Schamber-Reis and collaborators (Schamber-Reis et al. 2012) have clearly demonstrated that in *T. cruzi* the overexpression of the DNA polymerase beta enzyme displayed reduced levels of 8oxoG in kDNA and an increased survival after treatment with H_2_O_2_ when compared to control cells also showing its involvement in kinetoplast DNA replication and repair of oxidative lesions.

We decided, based on these data, to search for gene coding for proteins of the AP endonuclease family in *L. amazonensis* and evaluate their ability to repair damages caused by menadione an oxidative generator agent and also to 2-AF, an alkylating agent, in *E. coli* mutants strains for members of the AP endonuclease family.

*E. coli* DH5-α bacterial strain (Gibco® Life Technologies, MD, USA), *E. coli* mutant strain BW 535 (*nfo* ^-^, *xth* ^-^, *nth* ^-^), kindly provided by Dr Bernard Weiss (Georgia, USA) were used for functional complementation assays. *L. amazonenis* promastigotes (WHOM/BR/75/JOSEFA) were used for DNA extraction.

The following primers were employed in the PCR reactions for the amplification of the entire coding sequence of *L. amazonensis* gene homologous to the *L. major* AP endonuclease *(lmap*, GenBank U92487, 1344 bp): Lamap2-F Bg 5’-TCCAGATCTATGGCCTCGAAGCGATGCC-3’ and Lamap2-R Bg 5’ CCAGATCTTCATGGGTGTCGCATCCACAT-3 ‘. PCR reactions carried out with Taq DNA polymerase (Gibco BRL, MD, USA) were performed according to the manufacturer’s specifications, 2 mM of sense primer (Lamap2F - Bg) and 2 mM of antisense primer (Lamap2R-Bg), 200 ng of genomic DNA or from 1-2.5 ng of previously amplified PCR product. PCR reactions comprised a total of 30 cycles of annealing, denaturation and extension.

PCR products purified by the illustra GFX PCR DNA and Gel Band Purification Kit (GE Healthcare, Buckinghamshire, UK) were used for cloning into TOPO TA Cloning ® vector (pCR2.1) from Invitrogen Corporation. Each ligation reaction used 4 μL of the PCR product, equivalent to 400 ng, 1 μL of salt solution (1.2 M NaCl, 0.06 M MgCl_2_) diluted 4x, 1 μL of TOPO vector and sufficient Milli Q water to complete a final volume of 16 μL. The reagents were incubated for 30 min at room temperature and used to transform *E. coli* electrocompetent TOP10 cells accordingly to the manufacturer instructions. For automated DNA sequencing, extraction of plasmid DNA was performed from sixteen clones in 96-well microplates. About 300 ng of DNA and 2.5 pmol of primers were used in sequencing reactions with the DYEnamic ET Dye Terminator Kit (Amersham Biosciences, Amersham, UK) combined with the F-M13 primers (M13 universal 5’-GTAAAACGACGGCCAGT-3’) and M13 -R (5’-M13 reverse CAGGAAACAGCTATGAC-3‘), allowing the extension from the primers and vector-Lamap2F-Bg and Lamap2R-Bg specific for *lamap* gene*.* Automated sequencing was performed on a MegaBace 1000 sequencer (GE Healthcare). The files obtained in FASTA format corresponding to the clones were used for contig assembly by the Cap3 Sequence Assembly Program hosted at http://doua.prabi.fr/software/cap3 (Huang & Madan 1999). The resulting contig was translated by the tool EMBOSS Transeq at ebi.ac.uk/Tools/st/emboss_transeq/. Afterwards, the amino acid (aa) sequences obtained were searched against GenBank with blastx and the sequences with the highest value were selected and subjected to multiple sequence alignment using the ClustalX2.1 software (Larkin et al. 2007). The resulting FASTA file was exported to BioEdit (Hall 1999) and used for the final alignment comparing the potential LAMAP protein with sequences representing other members of the AP endonuclease family. We have assumed 60% of aa similarities as our cut-off limit for these analyses.

FASTA files from an alignment of eight exonucleases/endonucleases/phosphatases (EEP) superfamily aa sequences listed below were imported into MEGA version 6 (Tamura et al. 2013). Phylogenetic analyses were performed by Neighbor Joining with Kimura 2 Parameter. The consensus tree was obtained after bootstrap analysis with 1000 replications of the following aa sequences: *L. amazonensis* LAMAP (GenBank:KP_269080.1), *L. mexicana* AP endonuclease (GenBank:XP_003873754.1), *L. major* apurinic/apyri midinic endonuclease-redox protein(GenBank:XP_ 001682147.1), *T. cruzi* AP endonuclease 1 (GenBank: AGT41676.1), *Homo sapiens* AP endonuclease 1 (GenBank:P27695.2), *E. coli* EXO III (GenBank:|WP_ 000673937.1), *E. coli* ENDO III (GenBank:WP_0215775 34.1), *E. coli* ENDO IV (GenBank:NP_754582.1).

BW535, BW535pLamap and AB1157 (wild type *E.coli*) strains were submitted to challenge with an oxidative stress inducing agent known as menadione (MD) after the desired optical density of 0.5 U at 600 nm was reached. MD concentrations ranging from 0.5 mM-10 mM were used for the assay. Incubations were performed for 1 h at 37ºC using cupric chloride (5µM/mL) as catalyser. Samples were then diluted to 10^6^ cfu and 100 µL aliquots were plated on LB agar containing 100 µg/mL ampicillin (Sigma-Aldrich, MO, USA) and 40 µg/mL kanamycin (Gibco^®^ Life Technologies, MD, USA) for the BW535pLamap strain while the triple null mutant was incubated with kanamycin and the wild type *E. coli* strain AB1157 was plated in semi solid medium without antibiotics. Plates were incubated at 37ºC for 16-24 h. After this period colonies grown on plates were counted. Experiments were performed in triplicates. *E. coli* BW535 strain and BW535pLamap strain were also submitted to AF treatment where its concentration ranged from 0.97-1000 nM. All data including the mean Standard error media were generated using the Graph Pad Prism5 software (graphpad.com/scientific-software/prism/) and statistics analyses between the groups were performed with the one-way analysis of variance (ANOVA).

*L. amazonensis lamap* gene was amplified as a product of approximately 1400 bp, as expected. This amplicon was cloned into the pCR2.1 and sequenced, revealing a gene fragment of 1302bp. The results obtained with tblastx searches against GenBank showed that the most significant homologies were for *Leishmania* sp. proteins belonging to the family of AP endonucleases.

The putative AP endonuclease LAMAP displayed 99% (405/410) aa identity as well as the same level of positive amino acids (aas) corresponding to positions 38-447 of the *L. mexicana* AP endonuclease protein (GenBank: XP_003873754.1), 92% (376/410) aa identity and 386/410 (94%) positive aas corresponding also to positions 38-447 of the *L. major* AP endonuclease protein (GenBank: XP_001682147). The initial 37 N-terminal aas missing in LAMAP may not impact on its function as the main motifs involved at AP binding site, catalytic activities, DNA, metal or phosphate binding sites characterisitics of the Ape1-like_AP-endo (EEP superfamily of proteins) are present. New ongoing PCR and DNA sequencing strategies aiming the completeness of the *lamap* gene including its flanking regions might better address this issue.

The LAMAP ORF shows the motif LCLQETK which is characteristic of these enzymes (Barzilay & Hickson 1995, Mol et al. 1995, Pérez et al. 1999, Vidal et al. 2007) and is well conserved in the AP endonucleases of various organisms already analysed (Fig. 1A). This motif appeared in all eukaryotic repair proteins used in alignment as well as in EXO III from *E. coli* (LQETK). As previously reported by Pérez and collaborators for *L. major* (Pérez et al. 1999), the probable nuclear localisation signals for this class of proteins is also present in LAMAP (Fig. 1A). Phylogenetic analyses carried out with the *lamap* ORF resulted in a homogeneous tree with a topology that distinctly grouped the AP endonucleases of *Leishmania* spp from other trypanosomatids or AP endonucleases from human or *E. coli* (Fig. 1B). The corresponding similarity values observed were: 98% for *L. major*, *L. amazonensis* LAMAP AP1 and for *L. mexicana*. *T. cruzi* AP endonuclease (TCAP) was observed in another branch as well as the human apurinic endonuclease. The *E. coli* EXO III enzyme originated another branch that might represent the ancestor from which all the aa sequences previously reported above derived from. Lastly, the *E. coli* ENDO III and the *E. coli* ENDO IV are in two distinct branches, more distantly related to the AP included in this study.

**Fig. 1:**
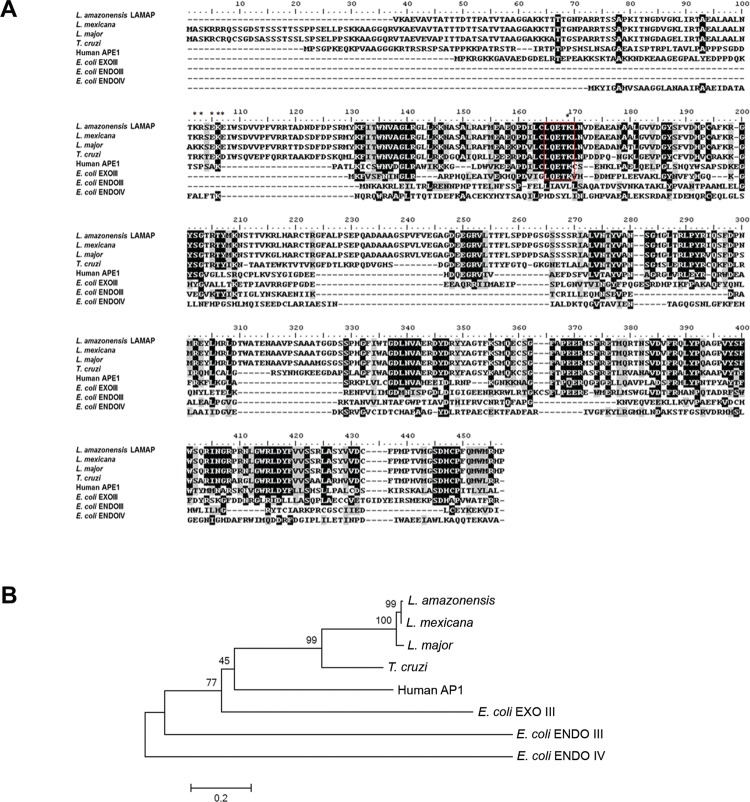
: LAMAP harbors conserved region domains of the AP endonuclease superfamily. (A) Multiple amino acid (aa) sequences alignment of members of the AP endonuclease superfamily from *Escherichia coli* and human (AP1) and corresponding sequences found in trypanosomatids after BLAST using the *Leishmania amazonensis* putative AP endonuclease (LAMAP) sequence as template. Identical amino acids are highlighted in black and similar in gray. The main residue block of aa presented in all organisms aligned (LQETK) is highlighted in red. Asterisks indicate residues responsible for nuclear localisation signals. (B) Phylogenetic tree constructed by neighbor-joining of exonucleases/endonucleases/phosphatases superfamily aa sequences. ENDO III: Endonuclease III. ENDO IV: Endonuclease IV. EXO III: Exonuclease III. Human AP1: human AP Endonuclease 1. Bootstrap values are displayed on each tree branch.

In experiments carried out using MD, we observed the ability of the *L. amazonensis* AP endonuclease enzyme (LAMAP) to restore *E. coli* viability after transformation with pCR2.1.Lamap plasmid (Fig. 2A). Without complementation, *E. coli* triple mutant for EXO III, ENDO III and ENDO IV BER enzymes presents high sensitivity to MD (Fig. 2A), and was unable to survive under concentrations up to 500 µM, while wild type *E. coli* AB1157 and pCR2.1.Lamap transformed strain BW535 presented survival even with concentrations up to 10 mM (Fig. 2A). Assays performed with AMF also suggest the ability of LAMAP to increase survival of triple mutant *E. coli* after transformation with pLamap evidencing its important role in DNA repair (Fig. 2B). Several studies have shown that among the defense mechanisms used by phagocytes against ingested organisms the production of reactive oxygen species represents one of the most important events. This phenomenon, known as respiratory burst, leads to DNA damage of phagocyted parasites, generating serious mutagenic and cytotoxic lesions, which often lead to cell death. It is well documented in the literature that parasites of the *Leishmania* genus have high sensitivity to different ROS such as H_2_O_2_ and that the production of these reactive species is configured as one of the main mechanisms of intracellular killing of amastigotes (Mittra et al. 2013). However, it is known that survival against these agents is an important virulence factor of *Leishmania* parasites and they possess resistance mechanisms to cope with ROS. Some of these mechanisms involve enzymes associated with the repair of DNA damages induced by substances described above (Wilson et al. 1994). Formation of abasic sites (apurinic and apyrimidinic) represents the main DNA lesion caused by ROS, with mutagenic and cytotoxic properties that must undergo efficient repair process in order to ensure viability of the injured organism. Several studies have demonstrated the important role of AP endonuclease in the repair of abasic sites (Wilson III & Barsky 2001) and mutant organisms lacking genes for members of this protein family show loss in viability after been submitted to different concentrations of oxidative and alkylating agents (Demple & Harrison 1994).

**Fig. 2:**
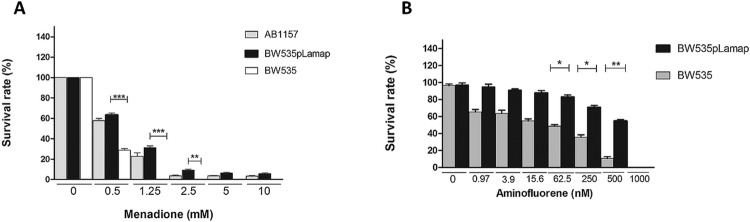
: LAMAP restores DNA repair activity in *Escherichia coli* Base Excision Repair deficient strains. (A) *E. coli* wild type strain AB1157 (gray column) triple null mutant BW535 (white column) and triple mutant transformed with pLamap (black column) challenged with increasing concentrations of menadione. (B) *E. coli* triple null mutant BW535 (gray column) and triple mutant transformed with pLamap (black column) challenged with increasing concentrations of aminofluorene. ***p > 0.001; **p > 0.01; *p > 0.05.

Despite the apparent importance of this class of enzymes, there are few studies on trypanosomes, including the genus *Leishmania*, which seem to be highly dependent on this repair pathway to remain viable in phagolysosomes. Pérez and co-workers (Pérez et al. 1999) have identified AP endonuclease genes in *L. major* and *T. cruzi* that confer resistance to oxidative agents when inserted into DNA repair deficient *E. coli* strains, which were called *lmap* in *L. major* and *TCAP* in *T. cruzi*.

Functional complementation assays using *E. coli* mutants strains for genes encoding the major families of these repair enzymes are often used to evaluate the biological activity of AP endonucleases (Demple & Harrison 1994). Perez and colleagues after complementation studies in mutant strains BW 286 (*xth* ^*-*^) and BW 528 (*xth* ^*-*^, *nfo* ^*-*^) demonstrated significant activity in endonuclease genes of *T. cruzi and L. major*, which corroborates with our data (Pérez et al. 1999).

When evaluating our data concerning the complementation assays, we observed that significant and meaningful results were obtained only with the triple mutant strain that lacks EXO III, IV ENDO, ENDO III enzyme activities. This finding is quite interesting because it shows us the efficiency of the *lamap* gene to repair both DNA injuries induced by alkylating and oxidative agents (Fig. 2B). Reports in the literature suggest that the enzyme Endo IV, which is responsible for about 5% of repair activity in *E. coli*, only has its activity observed in bacteria where EXO III is silenced, since EXO III is the main repair enzyme in *E. coli* and accounts for 95% of the activity of AP endonuclease (Wilson III & Barsky 2001).

Although well conserved between species, these repair enzymes have different biological characteristics. It seems that a similar situation occurs with the enzyme Endo IV when compared to EXO III. In our results the activity of the protein encoded by the *lamap* gene was better observed when none of the *E. coli* endogenous known AP-sites repairing enzymes were active.

Taken together, these data suggest that the repair activity of the *lamap* gene is closer to EXO III activity and can be better observed when this enzyme is not performing its normal function. Therefore, we believe that the *lamap* gene can be considered a candidate member of the AP endonucleases family in *L*. *amazonensis*. In this regard, a recent work by Sepúlveda and collaborators (Sepúlveda et al. 2014) also demonstrated by multiple aa sequence alignments that AP endonucleases from human (APE1, APE2), *Schizosaccharomyces pombe* (Apn2p) and *T. cruzi* (TcAP1, TcAP2) present conserved domains critical for AP binding and catalysis. LAMAP sequence presents all these residues, corroborating the idea that it corresponds to an AP Endonuclease.

While in the online database all *Leishmania* species with whole genomes sequenced present the AP endonucleases sequences, some of them are identified as putative genes. Although the *L. amazonensis* genome has been recently sequenced (Real et al. 2013) no functional annotation has been found for an AP endonuclease gene. One important approach to fill in this gap is represented by trypanosomatid comparative genomic studies that might bring contributions for the understanding of its biology and host-parasite interactions combining gene expression and functional genomics data resulting in potential new chemotherapeutical and vaccine targets (Teixeira et al. 2012).

The description and functional assays carried out in this work intend to collaborate in filling gaps such as this and suggest new approaches for the study of *Leishmania* host/pathogen interactions, since there are still few treatments available which are also considerably toxic. We believe that differences observed in the leishmanial AP Endonucleases at the aa level could be exploited for the design of new chemotherapeutic agents. In this regards it is worth to mention the review of Genois and co-workers (Genois et al. 2014) that clearly states that there is an interplay between DNA repair pathways and drug resistance mechanisms in trypanosomatids. One other relevant outcome of studies like this may also be to point out to the differences observed in the structure of leishmanial AP Endonucleases, particularly variations in nucleotide sequences that could be used as new typing tools.

Our results indicate that the *lamap* gene upon reestablishing the survival of BER deficient *E. coli* strains may also contribute to the intracellular persistence of *L. amazonensis* on its mammalian host cells.
